# Synthesis and Characterization of Thiol-Functionalized Polynorbornene Dicarboximides for Heavy Metal Adsorption from Aqueous Solution

**DOI:** 10.3390/polym14122344

**Published:** 2022-06-09

**Authors:** Alejandro Onchi, Carlos Corona-García, Arlette A. Santiago, Mohamed Abatal, Tania E. Soto, Ismeli Alfonso, Joel Vargas

**Affiliations:** 1 Instituto de Investigaciones en Materiales, Unidad Morelia, Universidad Nacional Autónoma de México, Antigua Carretera a Pátzcuaro No. 8701, Col. Ex Hacienda de San José de la Huerta, C.P. 58190 Morelia, Michoacán, Mexico; alejandro.onchi@gmail.com (A.O.); carcor93@gmail.com (C.C.-G.); tania@iim.unam.mx (T.E.S.); ialfonso@iim.unam.mx (I.A.); 2 Escuela Nacional de Estudios Superiores, Unidad Morelia, Universidad Nacional Autónoma de México, Antigua Carretera a Pátzcuaro No. 8701, Col. Ex Hacienda de San José de la Huerta, C.P. 58190 Morelia, Michoacán, Mexico; arlette_santiago@enesmorelia.unam.mx; 3 Facultad de Ingeniería, Universidad Autónoma del Carmen, Avenida Central S/N Esq. con Fracc. Mundo Maya, C.P. 24115 Ciudad del Carmen, Campeche, Mexico; mabatal@pampano.unacar.mx

**Keywords:** ROMP, thiol-functionalized polymer, polynorbornene dicarboximide, heavy metal adsorption, lead, cadmium, nickel

## Abstract

The contamination of water resources with heavy metals is a very serious concern that demands prompt and effective attention due to the serious health risks caused by these contaminants. The synthesis and ring-opening metathesis polymerization (ROMP) of norbornene dicarboximides bearing thiol pendant groups, specifically, *N*-4-thiophenyl-*exo*-norbornene-5,6-dicarboximide (**1a**), *N*-4-(methylthio)phenyl-*exo*-norbornene-5,6-dicarboximide (**1b**) and *N*-4-(trifluoromethylthio)phenyl-*exo*-norbornene-5,6-dicarboximide (**1c**), as well as their assessment for the removal of heavy metals from aqueous systems, is addressed in this work. The polymers were characterized by NMR, SEM and TGA, among others. Single and multicomponent aqueous solutions of Pb^2+^, Cd^2+^ and Ni^2+^ were employed to perform both kinetic and isothermal adsorption studies taking into account several experimental parameters, for instance, the initial metal concentration, the contact time and the mass of the polymer. In general, the adsorption kinetic data fit the pseudo-second-order model more efficiently, while the adsorption isotherms fit the Freundlich and Langmuir models. The maximum metal uptakes were 53.7 mg/g for Pb^2+^, 43.8 mg/g for Cd^2+^ and 29.1 mg/g for Ni^2+^ in the SH-bearing polymer **2a**, 46.4 mg/g for Pb^2+^, 32.9 mg/g for Cd^2+^ and 27.1 mg/g for Ni^2+^ in the SCH_3_-bearing polymer **2b** and 40.3 mg/g for Pb^2+^, 35.9 mg/g for Cd^2+^ and 27.8 mg/g for Ni^2+^ in the SCF_3_-bearing polymer **2c**, correspondingly. The better performance of polymer **2a** for the metal uptake was ascribed to the lower steric hindrance and higher hydrophilicity imparted by –SH groups to the polymer. The results show that these thiol-functionalized polymers are effective adsorbents of heavy metal ions from aqueous media.

## 1. Introduction

In the last decades, there has been a concerning rise in the number of heavy metals found in affluents due to the steady rise of industrial activities [1,2]. The pollution attributed to the industrial development is present in different ways, and among all the activities, metal industry for construction, battery manufacturing, mining and refining processes are the prime sources of releasing heavy metals to the environment, such as Pb^2+^, Cd^2+^ and Ni^2+^ [3,4]. The presence of these contaminants is known to be a serious harm to human health, besides being a considerable hazard to animals in the affected area. There are several techniques that are known to be effective in the elimination of heavy metals in aqueous media, for instance, ion exchange, osmosis, chemical precipitation and adsorption, among others [5,6,7]. In fact, the development and improvement of these and other new techniques are of the utmost importance for the urgent remediation of the environment.

Adsorption is a very interesting method due to its adaptability, good performance and the availability of a large number of materials with adsorption capacities [8,9]. In this sense, polymers have shown high effectiveness in the adsorption of heavy metals from aqueous systems due to their easy chemical functionalization, which allows adjusting the affinity towards heavy metal ions [10,11,12,13]. Norbornene-based monomers are readily functionalized, making the preparation of systematic series of norbornene derivatives an attainable goal [14]. Additionally, ring-opening metathesis polymerization (ROMP) is a useful tool for preparing and functionalizing polymers with outstanding physical and chemical integrity that can withstand harsh environments [15,16,17]. Furthermore, the information at hand points out that using functionalized ROMP-prepared polymers in adsorption processes is a fairly viable option for capturing Pb^2+^, Cd^2+^, Ni^2+^, Cr^3+^ and Cr^6+^ from aqueous systems [18,19]. 

Among the many functional group options used to endow materials with adsorption properties, thiols have drawn much attention and are well known for their ability to coordinate with divalent ions and have already been used successfully in the treatment of poisoning by Pb, Hg and As [20,21,22]. Since the ROMP affords high molecular weight polymers in high yields [23,24], ROMP-prepared thiol-containing polymers are expected to show high degrees of thiol moieties which in turn will likely result in high adsorption capacities. Based on the latter, ROMP-prepared polynorbornene derivatives could be used in the development of adsorbents bearing thiol groups as coordination points in order to tune the heavy metal adsorption capacity.

Hence, in this work, we carried out the synthesis and ROMP of three thiol-containing norbornene dicarboximide monomers along with the assessment of these polymers as adsorbents for capturing Pb^2+^, Cd^2+^ and Ni^2+^ from aqueous media. The physical properties, the thermal properties, and the surface morphology of the polymers are studied as well. As far as we know, the use of thiol-functionalized polynorbornene dicarboximides for heavy metal capture from water has not been reported, evidencing the necessity of exploring the assessment of this kind of macromolecular materials for such application.

## 2. Experimental Part

### 2.1. Characterization Techniques

The monomers and polymers obtained were characterized using the following techniques: Fourier-transform infrared spectroscopy (FT-IR), nuclear magnetic resonance (NMR), differential scanning calorimetry (DSC), thermomechanical analysis (TMA), thermogravimetric analysis (TGA) and X-ray diffraction (XRD). The FT-IR spectra were recorded in a Thermo Scientific Nicolet iS10 FT-IR spectrometer using an attenuated total reflectance (ATR) accessory with a diamond crystal. The final spectrum is an average of thirty-two spectra collected for each sample in a range of 4000–650 cm^−1^ at a spectral resolution of 4 cm^−1^. The ^1^H-NMR, ^13^C-NMR and ^19^F-NMR spectra were recorded on a Bruker Avance III HD at 400, 100 and 376 MHz, respectively. Tetramethylsilane (TMS) and hexafluorobenzene (HFB) were used as internal standards for the NMR analysis. The samples were measured in CDCl_3_ for **1a**, **1b**, **1c**, **2b** and **2c**, while the polymer **2a** was measured in DMF-d_7_. DSC was carried out in a SENSYS evo DSC, with samples encapsulated in standard aluminum DSC pans, at a scanning rate of 10 °C min^−1^ under a nitrogen atmosphere in a temperature range between 30 °C and 500 °C, and it was used for determining the glass transition temperature (*T_g_*) of the polymers. The TMA was carried out in a TA Instruments Thermomechanical Analyzer TMA Q400, at a rate of 10 °C min^−1^ under a nitrogen atmosphere to corroborate the *T_g_* values. TGA was conducted to determine the onset of decomposition temperature (*T_d_*) of the polymers. The samples, around 10 mg, were heated at a rate of 10 °C min^−1^ from 30 to 600 °C under a nitrogen atmosphere in a TA Instruments Thermogravimetric Analyzer TGA 5500. XRD measurements were performed in polymer films on a Bruker D2-Phaser 2nd Generation diffractometer between 7 and 70° 2θ, at 30 KV 10 mA, using CuK_α_ radiation (1.54 Å). The polymer density (*ρ*) was measured in film form in ethanol at room temperature using the analytical balance model Sartorius Quintix 124-1s by the flotation method.

The heavy metal adsorption capacity was calculated through a series of experiments using Pb^2+^, Cd^2+^ and Ni^2+^ aqueous solutions where PbCl_2_, CdCl_2_·2.5H_2_O y NiCl_2_·6H_2_O were used to prepare the respective solutions. For the study of the adsorption kinetics, 0.01 g of each polymer were put in contact with a 100-ppm solution for 5, 10, 15, 30, 60, 120, 180, 360, 720 and 1440 min, respectively. For the study of the adsorption isotherms, 0.01 g of each polymer were put in contact with a solution of 10, 20, 40, 60, 80, 100, 200, 300, 400 and 500 ppm for 24 h, respectively. For the study of the mass effect, 0.01, 0.02, 0.05, 0.08 and 0.10 g of each polymer were put in contact with a 100-ppm solution of each metal for 24 h. For the study of the multicomponent adsorption, a 100 ppm of ternary heavy metal solution using a 100-ppm solution of each metal ion was prepared, and 0.01 g of each polymer was put in contact with it for 24 h. The changes in heavy metal concentration in the solution after the contact with the polymers were quantified using atomic absorption spectroscopy (AAS) in a Thermo Scientific iCE 3000 Series.

### 2.2. Reagents

The *exo*-norbornene-5,6-dicarboxylic anhydride (NDA) was obtained via the Diels-Alder cycloaddition reaction of maleic anhydride and cyclopentadiene in accordance with the literature [14]. 4-Aminothiophenol, 4-(methylthio)aniline, 4-(trifluoromethylthio)aniline and tricyclohexylphosphine [1,3-bis(2,4,6-trimethylphenyl)-4,5-dihydroimidazol-2-ylidene][benzylidene] ruthenium dichloride (2nd Generation Grubbs catalyst) were employed as they were received. Lead(II) chloride (PbCl_2_), cadmium(II) chloride hemi(pentahydrate) (CdCl_2_·2.5H_2_O), nickel(II) chloride hexahydrate (NiCl_2_·6H_2_O), chloroform and methanol were employed as they were received. Dichloromethane and 1,2-dichloroethane were dried over anhydrous CaCl_2_, then distilled over CaH_2_ and used as solvents. All reagents were purchased from Merck Sigma-Aldrich.

### 2.3. Synthesis and Characterization of Monomers

#### 2.3.1. Monomer *N*-4-thiophenyl-*exo*-norbornene-5,6-dicarboximide (**1a**)

**Scheme 1:**
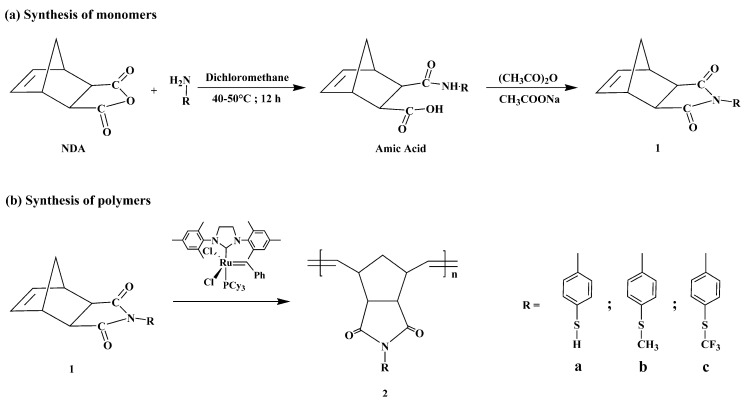
(**a**) Synthesis of the thiol-functionalized monomers and (**b**) synthesis of the thiol-containing polymers via ROMP.

In total, 30 mL of dichloromethane were used to dissolve 0.30 g (0.0023 mol) of 4-aminothiophenol and 0.39 g (0.0023 mol) of NDA. The solution was stirred for 12 h at 60 °C to give an amic acid. Next, the amic acid was filtered and dissolved in 30 mL of (CH_3_CO)_2_O, then 0.50 g of CH_3_COONa were added to the mixture and kept with constant stirring for 12 h at 70 °C. The product precipitated on pouring the reaction mixture into iced water and was then filtered. Finally, the monomer was recrystallized twice from a toluene:hexane (1:1) solution and dried at 50 °C for 12 h under vacuum. The pure monomer was obtained in 72% yield. Melting point (MP): 166–168 °C (Scheme 1a).

FT-IR (powder, cm^−1^): υ 3070 (C=C–H aromatic (ar.) stretching (str.)), 2990 (C–H asymmetric (asym.) str.), 2885 (C–H symmetric (sym.) str.), 1774 (C=O), 1697 (C=O), 1492 (C=C str.), 1373 (C–N) and 684 (C–S).

^1^H-NMR (400 MHz, CDCl_3_, ppm): δ 7.55–7.37 (4H, m, ar.), 6.39 (2H, s), 3.42 (2H, s), 2.88 (2H, s), 2.45 (1H, s), 1.64 (1H, m) and 1.46 (1H, m).

^13^C-NMR (100 MHz, CDCl_3_, ppm): δ 176.6 (C=O), 138.0 (C=C), 126.7 (C–S) 47.8, 45.9 and 43.0.

#### 2.3.2. Monomer *N*-4-(methylthio)phenyl-*exo*-norbornene-5,6-dicarboximide (**1b**)

In total, 30 mL of dichloromethane were used to dissolve 0.30 g (0.0021 mol) of 4-(methylthio)aniline and 0.35 g (0.0021 mol) of NDA. The solution was stirred for 12 h at 60 °C to give an amic acid. Next, the amic acid was filtered and dissolved in 30 mL of (CH_3_CO)_2_O, then 0.50 g of CH_3_COONa were added to the mixture and kept with constant stirring for 12 h at 70 °C. The product precipitated on pouring the reaction mixture into iced water and was then filtered. Finally, the monomer was recrystallized twice from ethanol and dried at 50 °C for 12 h under vacuum. The pure monomer was obtained in 61% yield. Melting point (MP): 164–166 °C (Scheme 1a).

FT-IR (powder, cm^−1^): υ 3073 (C=C–H ar. str.), 2980 (C–H asym. str.), 2945 (C–H sym. str.), 1773 (C=O), 1698 (C=O), 1493 (C=C str.), 1438 (C–H), 1385 (C–N) and 681 (C–S).

^1^H-NMR (400 MHz, CDCl_3_, ppm): δ 7.36–7.18 (4H, m, ar.), 6.36 (2H, s), 3.42 (2H, s), 2.87 (2H, s), 2.51 (3H, s), 1.65 (1H, m) and 1.47 (1H, m).

^13^C-NMR (100 MHz, CDCl_3_, ppm): δ 177.0 (C=O), 138.0 (C=C), 125.3 (C–S), 47.8, 45.8 and 43.0.

#### 2.3.3. Monomer *N*-4-(trifluoromethylthio)phenyl-*exo*-norbornene-5,6-dicarboximide (**1c**)

In total, 30 mL of dichloromethane were used to dissolve 0.20 g (0.0010 mol) of 4-(trifluoromethylthio)aniline and 0.16 g (0.0010 mol) of NDA. The solution was stirred for 12 h at 60 °C to give an amic acid. Next, the amic acid was filtered and dissolved in 30 mL of (CH_3_CO)_2_O, then 0.50 g of CH_3_COONa were added to the mixture and kept with constant stirring for 12 h at 70 °C. The product precipitated on pouring the reaction mixture into iced water and was then filtered. Finally, the monomer was recrystallized twice from hexane and dried at 50 °C for 12 h under vacuum. The pure monomer was obtained in 49% yield. Melting point (MP): 157–159 °C (Scheme 1a).

FT-IR (powder, cm^−1^): υ 3068 (C=C–H ar. str.), 2988 (C–H asym. str.), 2970 (C–H sym. str.), 1775 (C=O), 1706 (C=O), 1493 (C=C str.), 1382 (C–N), 1109 (C–F) and 683 (C–S).

^1^H-NMR (400 MHz, CDCl_3_, ppm): δ 7.77–7.38 (4H, m, ar.), 6.38 (2H, s), 3.42 (2H, s), 2.88 (2H, s), 1.65 (1H, m) and 1.43 (1H, m).

^13^C-NMR (100 MHz, CDCl_3_, ppm): δ 172.5 (C=O), 138.0 (C=C), 47.8, 45.8 and 43.0.

^19^F-NMR: (376 MHz, CDCl_3_, ppm): δ −44.75 (CF_3_).

### 2.4. ROMP of the Thiol-Functionalized Monomers

Monomer polymerizations were carried out in 25 mL round bottom flasks under N_2_ atmosphere. C_2_H_5_OCH=CH_2_ was employed as an inhibitor in the final stage. The polymers were obtained by pouring the reaction solution into 60 mL of acidified methanol under constant stirring at 50 °C, precipitating in the form of white fibers. The resulting polymers were collected by filtration, then dissolved in chloroform and poured again into hot stirring methanol for being purified. Next, the products were dried for 12 h at 50 °C under vacuum.

#### 2.4.1. Synthesis and Characterization of Poly(*N*-4-thiophenyl-*exo*-norbornene-5,6-dicarboximide) (**2a**)

In total, 10 mL of 1,2-dichloroethane were employed to dissolve 0.2 g (0.73 mmol) of monomer **1a** and 6.1 × 10^−4^ g (7.1 × 10^−4^ mmol) of Grubbs catalyst. The polymerization reaction was stirred at room temperature for 2 h (Scheme 1b). The thiol-functionalized polymer **2a** was soluble in *N*,*N*-dimethylformamide (DMF) and 1,2-dichloroethane.

FT-IR (thin film, cm^−1^): ʋ 3472 (C=C–H ar. str.), 2922 (C–H asym. str.), 2852 (C–H sym. str.), 1776 (C=O), 1703 (C=O), 1494 (C=C str.), 1367 (C–N) and 659 (C–S).

^1^H-NMR (400 MHz, DMF-d_7_, ppm): δ 7.61−7.28 (4H, m, ar.), 5.87 (1H, s, *trans*), 5.63 (1H, s, *cis*), 3.45 (1H, s), 3.18 (2H, s), 2.76 (1H, s), 2.46 (1H, s), 2.18 (1H, s) and 1.69 (1H, s).

^13^C-NMR (100 MHz, DMF-d_7_, ppm): δ 176.6 (C=O), 138.0 (C=C), 134.8, 126.7, 77.3, 47.8, 45.9 and 43.0.

#### 2.4.2. Synthesis and Characterization of Poly(*N*-4-(methylthio)phenyl-*exo*-norbornene-5,6-dicarboximide) (**2b**)

In total, 2 mL of 1,2-dichloroethane were employed to dissolve 0.2 g (0.51 mmol) of monomer **1b** and 7.6 × 10^−4^ g (8.9 × 10^−4^ mmol) of Grubbs catalyst. The polymerization reaction was stirred at 45 °C for 2 h (Scheme 1b). The thiol-functionalized polymer **2b** was soluble in *N*,*N*-dimethylformamide (DMF), chloroform and 1,2-dichloroethane.

FT-IR (thin film, cm^−1^): ʋ 3627 (C=C–H ar. str.), 2919 (C–H asym. str.), 2855 (C–H sym. str.), 1773 (C=O), 1701 (C=O), 1495 (C=C str.), 1436 (C–H), 1373 (C–N) and 660 (C–S).

^1^H-NMR (400 MHz, CDCl_3_, ppm): δ 7.31−7.05 (4H, m, ar.), 5.78 (1H, s, *trans*), 5.52 (1H, s, *cis*), 3.48 (1H, s), 3.26 (2H, s), 2.83 (1H, s), 2.43 (3H, s), 2.09 (1H, s) and 1.60 (1H, s).

^13^C-NMR (100 MHz, CDCl_3_, ppm): δ 177.5 (C=O), 139.7 (C=C), 134.4, 127.0, 125.0, 77.9, 53.8, 51.4 and 46.9.

#### 2.4.3. Synthesis and Characterization of Poly(*N*-4-(trifluoromethylthio)phenyl-*exo*-norbornene-5,6-dicarboximide) (**2c**)

In total, 2 mL of 1,2-dichloroethane were employed to dissolve 0.2 g (0.58 mmol) of monomer **1c** and 9.5 × 10^−4^ g (1.1 × 10^−3^ mmol) of Grubbs catalyst. The polymerization reaction was stirred at 45 °C for 2 h (Scheme 1b). The thiol-functionalized polymer **2c** was soluble in *N*,*N*-dimethylformamide (DMF), chloroform and 1,2-dichloroethane.

FT-IR (thin film, cm^−1^): ʋ 3480 (C=C–H ar. str.), 2954 (C–H asym. str.), 2859 (C–H sym. str.), 1779 (C=O), 1707 (C=O), 1495 (C=C str.), 1366 (C–N), 1109 (C–F) and 659 (C–S).

^1^H-NMR (400 MHz, CDCl_3_, ppm): δ 7.77−7.19 (4H, m, ar.), 5.84 (1H, s, *trans*), 5.59 (1H, s, *cis*), 3.51 (1H, s), 3.22 (2H, s) 2.91 (1H, s), 2.12 (1H, s) and 1.61 (1H, s).

^13^C-NMR (100 MHz, CDCl_3_, ppm): δ 176.9 (C=O), 147.2 (C=C), 137.1, 134.5, 127.4, 124.8, 77.7, 54.0, 51.2 and 47.1.

^19^F-NMR: (376 MHz, CDCl_3_, ppm): δ −46.8 (CF_3_).

### 2.5. Membrane Preparation

Membranes were cast from polymeric 1,2-dichloroethane solutions (~8 wt%) at room temperature. The solution was filtered and poured onto a glass plate, and the solvent was allowed to evaporate slowly under a controlled 1,2-dichloroethane atmosphere. Then, the membranes were dried under a vacuum at 100 °C for 24 h. The average thickness of the films was around 50 μm. The prepared films were only used for FT-IR, XRD, thermal and density measurement purposes.

### 2.6. Heavy Metal Adsorption

Pb^2+^, Cd^2+^ and Ni^2+^ stock solutions of 1000 mg/L were prepared by dissolving PbCl_2_, CdCl_2_·2.5H_2_O and NiCl_2_·6H_2_O, respectively, in deionized water. Before each series of AAS measurements, the deionized water was first used as a blank to discard any heavy metal trace that could be in the remaining liquid. Subsequently, each heavy metal solution concentration was corroborated using AAS before the respective experiments.

### 2.7. Adsorption Kinetics

For the adsorption kinetics experiments, solutions of Pb^2+^, Cd^2+^ and Ni^2+^ with concentrations of 100 ppm were prepared, respectively. An amount of 0.01 g of each polymer was added, separately, to a 10 mL aliquot of individual heavy metal solution. The mixtures of each polymer with each solution were kept in constant stirring using a rotatory shaker for 5, 10, 15, 30, 60, 120, 180, 360, 720 and 1440 min. After a specific contact time, the samples were centrifuged at 3500 rpm for 10 min to separate the polymer from the solution. Then, AAS was used to measure the final solution concentration. The quantity of heavy metal adsorbed on the surface of each polymer at a specific time, denoted as *q_t_* (mg/g), was calculated employing Equation (1), knowing that *m* is the amount of polymer used for each sample (g), *V* is the solution volume (L), *C_0_* is the initial heavy metal concentration (mg/L) and *C_f_* is the final heavy metal concentration (mg/L).

*q_t_* = *V*(*C*_0_ − *C_f_*)/*m* (1)

To describe the adsorption kinetics of the respective metals on the polymer surface, pseudo-first and pseudo-second-order models were applied to the analysis of the experimental data. Both mathematical expressions were linearized and are shown in Equations (2) and (3), respectively.

log(*q_e_* − *q_t_*) = log(*q_e_*) − (*k_1_*/2.303)*t* (2)

*t*/*q_t_* = {(1/(*k_2_ q_e_*^2^)} + (1/*q_e_*)*t* (3)

*q_t_* (mg/g) is the concentration of the heavy metal at a time *t* (min) and *q_e_* (mg/g) is the concentration of the heavy metal in equilibrium. Likewise, *k_1_* (1/min) and *k_2_* (g/mg·min) are the pseudo-first and pseudo-second-order model equilibrium rate constants, respectively.

### 2.8. Isotherm Study

Regarding the isotherm studies, solutions of Pb^2+^, Cd^2+^ and Ni^2+^ were prepared with the following concentrations: 10, 20, 40, 60, 80, 100, 200, 300, 400 and 500 ppm, then, 0.01 g of each polymer was added to every solution separately and was kept with constant stirring in a rotatory shaker for 24 h. Once the contact time was reached, the samples were subjected to centrifugation for 10 min at 3500 rpm to separate the polymer from the solution. Then, AAS was used to measure the final solution concentration.

For the analysis of the experimental data, the Freundlich and Langmuir models were used to explain the equilibrium adsorption of the heavy metals on the polymeric surface. The equations of these models were linearized and are shown in Equations (4) and (5), respectively.

ln(*q_e_*) = ln(*K_F_*) + {(1/n) ln(*C_e_*)} (4)

1/*q_e_* = 1/*q_m_* + {1/(*q_m_K_L_*)}{1/*C_e_*} (5)

*q_m_* (mg/g) is the maximum concentration of heavy metal, *q_e_* (mg/g) is the equilibrium concentration of heavy metal and *C_e_* (mg/L) is the heavy metal concentration in the equilibrium of the solution. Likewise, *K_F_* (1/min) and *K_L_* (g/mg·min) are the Freundlich and Langmuir model equilibrium rate constants, respectively.

### 2.9. Mass Effect Study

The effect of the mass on the adsorption capacity was assessed by adding 0.01, 0.02, 0.05, 0.08 and 0.10 g of each polymer to 100 ppm solutions of Pb^2+^, Cd^2+^ and Ni^2+^, respectively. The polymer was left in contact with the solution and stirred for 24 h in a rotatory shaker; next, the samples were centrifuged at 3500× *g* rpm for 10 min to separate the polymer from the solution. Then, AAS was used to measure the final solution concentration.

### 2.10. Multicomponent Adsorption Study

For studying the adsorption capacity of the polymers in a multicomponent solution, a unique 100 ppm solution of Pb^2+^, Cd^2+^ and Ni^2+^ was prepared and 0.01 g of each polymer was put in contact, with constant stirring in a rotatory shaker, with the solution for 24 h. Once the contact time was reached, the samples were subjected to centrifugation for 10 min at 3500× *g* rpm to separate the polymer from the solution. Then, AAS was used to measure the final solution concentration.

## 3. Results and Discussions

**Figure 1:**
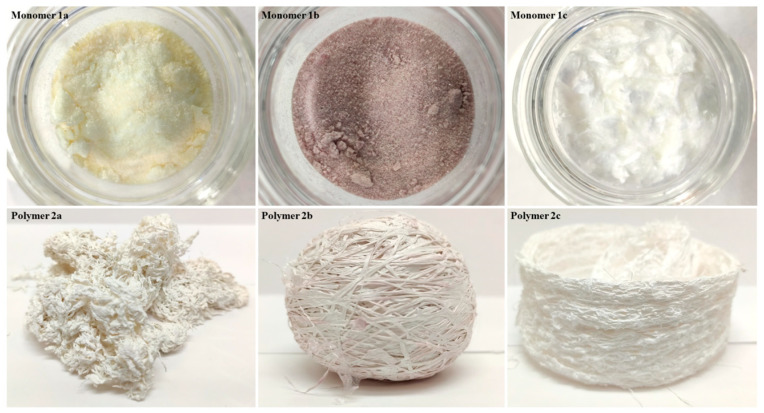
Photographic images of the thiol-functionalized norbornene dicarboximide monomers (**top**) and their corresponding ROMP-prepared raw polymers (**bottom**).

Monomers **1a**, **1b** and **1c** were prepared successfully in a two-step reaction in 72%, 61% and 49% yield, respectively. NDA reacted with 4-aminothiophenol, 4-(methylthio)aniline and 4-(trifluoromethylthio)aniline, respectively, to yield amic acids, which were dehydrated employing acetic anhydride and anhydrous sodium acetate to afford the corresponding thiol-functionalized imide monomers (Scheme 1). The melting points of the monomers were in the range of 166–168 °C, 164–166 °C and 157–159 °C for **1a**, **1b** and **1c**, respectively. Photographic images of the thiol-functionalized norbornene dicarboximide monomers synthesized in this study as well as their corresponding raw polymers prepared by ROMP are shown in Figure 1. It can be seen that the monomers exhibit different colorations depending on the thiol substituent attached to the aromatic ring being pale yellow for monomer **1a**, slightly violet for monomer **1b** and white for monomer **1c**. Despite the monomer coloration, all of them afforded white fibrous polymers capable of forming tough transparent films.

**Figure 2:**
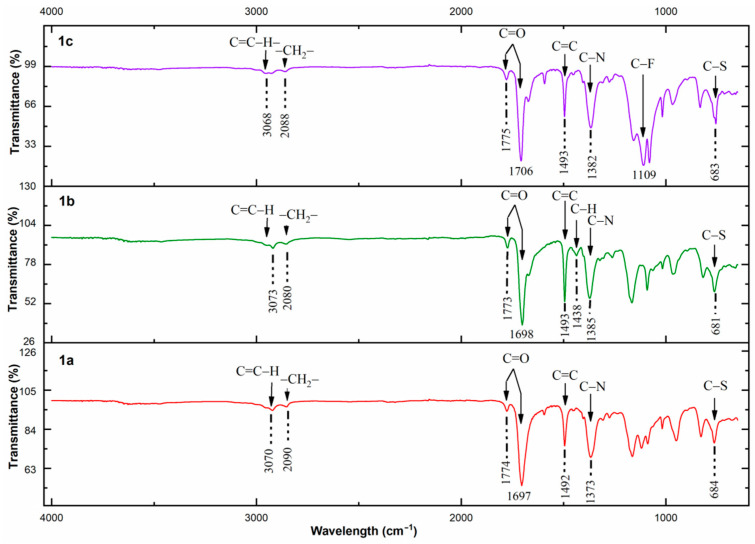
FT-IR spectra of the thiol-functionalized norbornene dicarboximide monomers.

The chemical structures of the thiol-functionalized norbornene dicarboximide monomers were confirmed by FT-IR spectroscopy and the spectra are shown in Figure 2. Spectra are very similar to each other with slight differences due to the thiol group substituents. In general, the following characteristic absorption bands can be observed: the absorption band of the H–C bond in the aromatic groups is seen at about 3070 cm^−1^; the bands of the C–H bond in the methylene groups corresponding to the antisymmetric and symmetric vibration modes are observed at 2990 and 2945 cm^−1^, respectively; likewise, the bands of the C=O bond associated to the antisymmetric and symmetric vibration modes are shown at 1774 and 1697 cm^−1^; correspondingly, the absorption band of the C=C bond related to the stretching mode is displayed at 1492 cm^−1^; the absorption band of the C–H bond in the methyl groups corresponding to the stretching mode is observed around 1438 cm^−1^; the band of the C–N bond attributed to the stretching mode is seen about 1382 cm^−1^; the band of the C–F bond assigned to the stretching mode was found around 1109 cm^−1^; the signal observed about 683 cm^−1^ is ascribed to the stretching vibration mode of the C–S bond.

**Figure 3:**
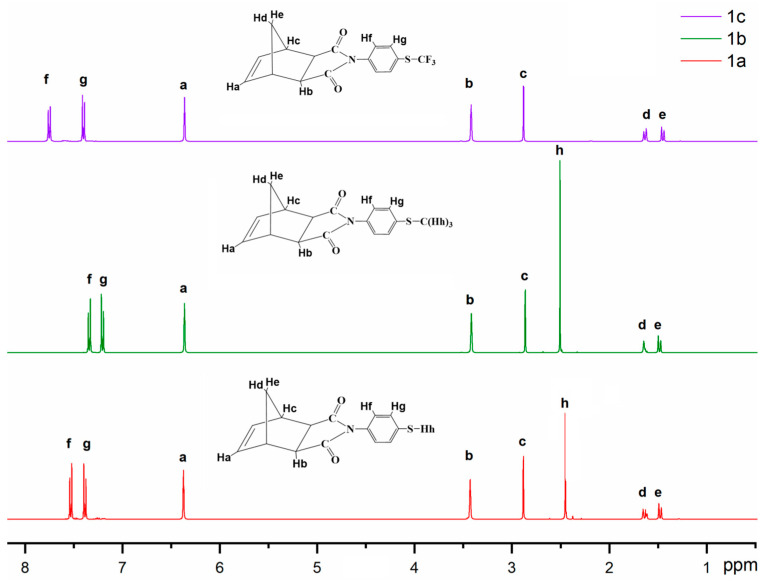
^1^H-NMR spectra of the thiol-functionalized norbornene dicarboximide monomers.

The monomer’s chemical structures were also corroborated by ^1^H-NMR, ^13^C-NMR and ^19^F-NMR. The ^1^H-NMR spectra of all monomers exhibited aromatic proton signals (H_f_ and H_g_) in the range of 7.61–7.18 ppm (Figure 3). The signals corresponding to the olefinic protons (H_a_) ranged from 6.39 to 6.36 ppm, while the signals associated with the protons of the –CH– groups (H_b_ and H_c_) were found in the range of 3.42–2.87 ppm. For all monomers, the signals ascribed to the methylene protons (H_d_ and H_e_) were seen ranging from 1.65 to 1.43 ppm. Finally, the signals associated with the protons of the thiol groups (H_h_) were observed at 2.45 ppm for monomer **1a** and at 2.51 ppm for monomer **1b**. The ^13^C-NMR spectra of the monomers showed characteristic signals for the carbon atoms in the C=O groups ranging between 172.5 and 177.0 ppm while the signals associated with the carbon atoms in the C=C groups were observed at 138.0 ppm for all monomers. Likewise, the ^19^F-NMR analysis indicated that fluorine atoms in the –CF_3_ groups of monomer **1c** are magnetically equivalent, leading to the appearance of one signal peak around −46.8 ppm.

**Figure 4:**
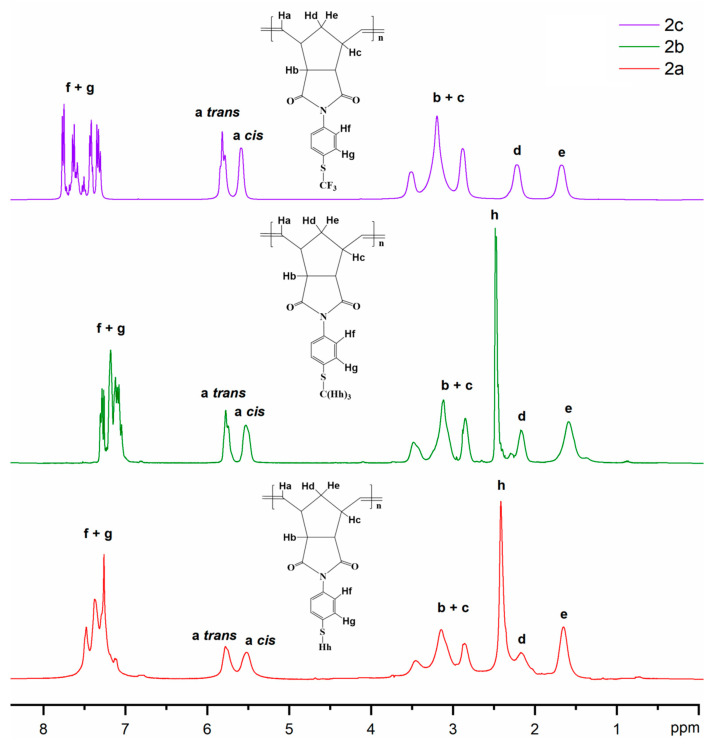
^1^H-NMR spectra of the polynorbornene dicarboximides bearing thiol pendant groups.

The polymers ^1^H-NMR spectra are shown in Figure 4. As it is seen, the ^1^H-NMR spectra of the polymers are very similar to those of the monomers. For all polymers, the signals attributed to the aromatic protons (H_f_ and H_g_) appeared between 7.77 and 7.05 ppm. In general, the olefinic proton signals of the monomers around δ = 6.38 ppm are substituted by new olefinic proton (H_a_) signals that arise in the range of δ = 5.87–5.78 ppm and 5.63–5.52 ppm, corresponding, respectively, to the *trans* and *cis* double bonds of the polymer backbone. The proton signals of the –CH– groups (H_b_ and H_c_) were observed in the range of 3.51–2.76 ppm. The signals associated with the methylene protons (H_d_ and H_e_) were seen ranging from 2.18 to 1.60 ppm, with slight signal overlapping arising in this area owing to the signals from the protons (H_h_) attached to the thiol substituents that appeared at 2.46 and 2.43 ppm for the polymers **2a** and **2b**, respectively.

**Figure 5:**
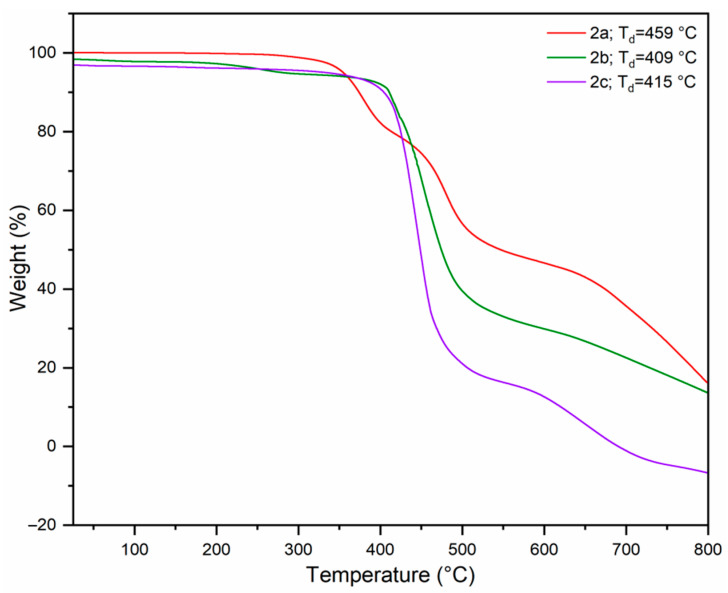
Thermal decomposition of the polymers bearing thiol moieties.

Table 1 summarizes the physical properties of the polymers bearing thiol pendant groups. The glass transition temperature (*T_g_*) was measured by DSC and the values were corroborated by TMA. The *T_g_* values obtained were 225 °C, 216 °C and 215 °C for the polymers **2a**, **2b** and **2c**, respectively. The decrease in the *T_g_* value for polymers **2b** and **2c** compared to that of **2a** is attributed to the substituent on the thiol group; since the methylthiol and trifluoromethylthiol groups introduce more free volume into the polymer chains, higher conformational mobility is achieved, which in turn lowers the *T_g_*. The polymer’s thermal stability was studied under a nitrogen atmosphere by TGA. The thermal decomposition curves, shown in Figure 5, were shifted by 2% from each other starting from that corresponding to the polymer bearing –SH groups, **2a**, to clearly track the decomposition profiles of all the thiol-functionalized polynorbornene dicarboximides. From Figure 5, it can be seen that all the thiol-functionalized polymers show the onset temperature for decomposition (*T_d_*) in the range of 409–459 °C, indicating that the norbornene dicarboximide monomers reported here yield polymers of relatively high thermal stability. The polymer density, *ρ*, was measured in film form by the flotation method at ambient conditions in ethanol [25]. In Table 1, it is noticed that polymer **2a** exhibits the higher density of all the polymers studied here, which could be associated with a higher polymer chain packing efficiency promoted by the thiol groups. In polymer **2b**, the presence of methylthiol groups causes greater steric hindrance than the thiol groups in polymer **2a**, which decreases the chain packing efficiency and, in turn, the polymer density. Likewise, the presence of bulky trifluoromethylthiol groups in polymer **2c** also diminishes the polymer packing efficiency, which leads to a lower density than that of polymer **2a**. 

**Figure 6:**
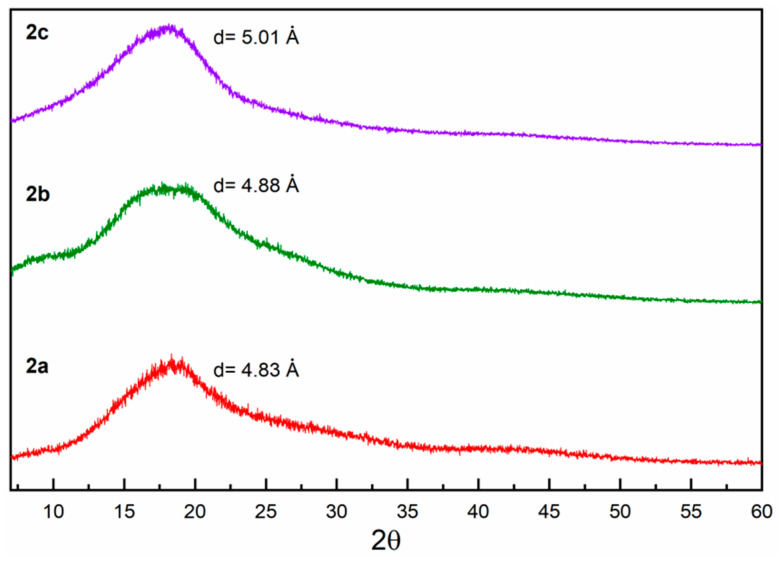
X-ray diffraction patterns of the polymers bearing thiol moieties.

XRD measurements carried out in films of the thiol-functionalized polynorbornene dicarboximide revealed amorphous polymers with no regions of crystallinity. The diffraction patterns showed a characteristic broad peak for polynorbornene dicarboximides with the highest intensity of reflection in the range of 17–20°, in the 2*θ* scale (Figure 6) [19]. Using Bragg’s equation, *nλ* = 2*d*sin*θ*, the *d*-spacing is determined at the maximum reflection angle of the amorphous curve [26]. This parameter is considered a measure of the mean intersegmental distance of the polymer chains and can be used to understand the effect of the different thiol substituents on the chain packing efficiency. In Table 1, it can be noticed that *d*-spacing decreases in the order **2c** > **2b** > **2a**, which suggests that the trifluoromethylthiol and methyilthiol groups exhibit higher steric hindrance than the thiol group. This trend in the *d*-spacing values correlates quite well with the values found for the density since both parameters are inversely affected by the chain packing efficiency.

### 3.1. Kinetics

**Figure 7:**
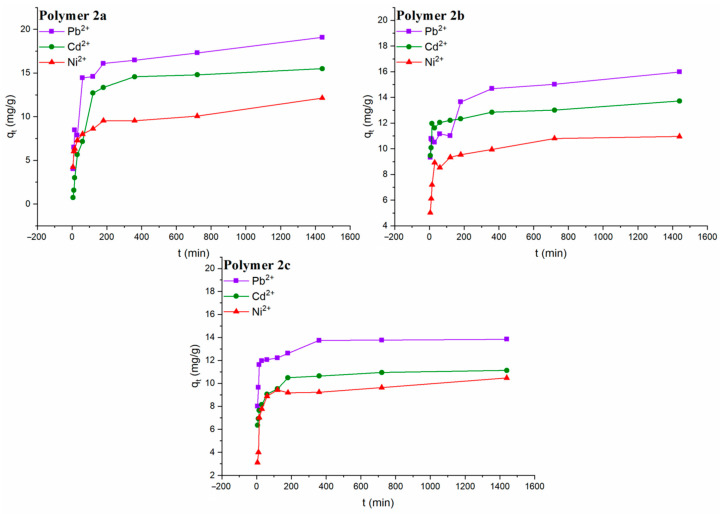
Amount of heavy metal uptaken by the polymers **2a**, **2b** and **2c** as a function of time (*t*).

The adsorption kinetics of the different polymers can be observed in Figure 7, where it is shown the adsorption capacity (*q_t_*) as a function of the time of contact (*t*). Due to a large number of available sites for coordination at the beginning of the experiment, it is possible to observe a rapid increase in the adsorption of the metallic ions up to 180 min; once the divalent ions start to occupy several adsorption sites, the adsorption rate slowly stabilized until equilibrium was reached. The high heavy metal concentration at the beginning of the experiment also can produce a concentration gradient that contributes to the fast adsorption in the early stages. It is also possible to observe a tendency in the quantity of heavy metal adsorbed for each polymer, where Pb^2+^ is the most adsorbed ion, followed by Cd^2+^ and finally Ni^2+^. It is seen that the higher the atomic number, the more probability of coordinating with the thiol group; thus, lead, having a higher atomic number than cadmium, is adsorbed in greater quantity. In the same way, cadmium is adsorbed in greater quantity than nickel.

The pseudo-first-order and pseudo-second-order models were applied to estimate the kinetic parameters and the theoretical adsorption capacity at equilibrium (*q_e, the_*); the data are presented in Table 2 with the maximum experimental adsorption capacity (*q_t, exp_*). The *q_t_*_, *exp*_ for the polymer **2a** was 19.07 mg/g for Pb^2+^, 15.49 mg/g for Cd^2+^ and 12.12 mg/g for Ni^2+^. The maximum experimental capacity for the polymer **2b** was 15.98 mg/g for Pb^2+^, 13.71 mg/g for Cd^2+^ and 10.95 mg/g for Ni^2+^. The maximum experimental capacity for the polymer **2c** was 13.84 mg/g for Pb^2+^, 11.12 mg/g for Cd^2+^ and 10.47 mg/g for Ni^2+^. The correlation coefficients (*R*^2^) allow us to determine which model is more appropriate for each type of adsorption. The *R*^2^ values point out that the pseudo-second-order model better describes the heavy metal adsorption on these polymers and that the theoretical maximum capacity of adsorption (*q_e, the_*) of this model fits better with the experimental maximum adsorption capacity (*q_t, exp_*) obtained in this study. This theoretical model suggests that the determining step for the three different polymers is not the mass transfer but rather the adsorption reaction. The velocity constants observed in the theoretical predictions allow us to know the rate at which the adsorption takes place, being Ni^2+^ the fastest ion to be adsorbed by polymer **2a**, Cd^2+^ is the fastest ion to be adsorbed by polymer **2b** and Pb^2+^ is the fastest ion to be adsorbed by polymer **2c**.

### 3.2. Isotherms

**Figure 8:**
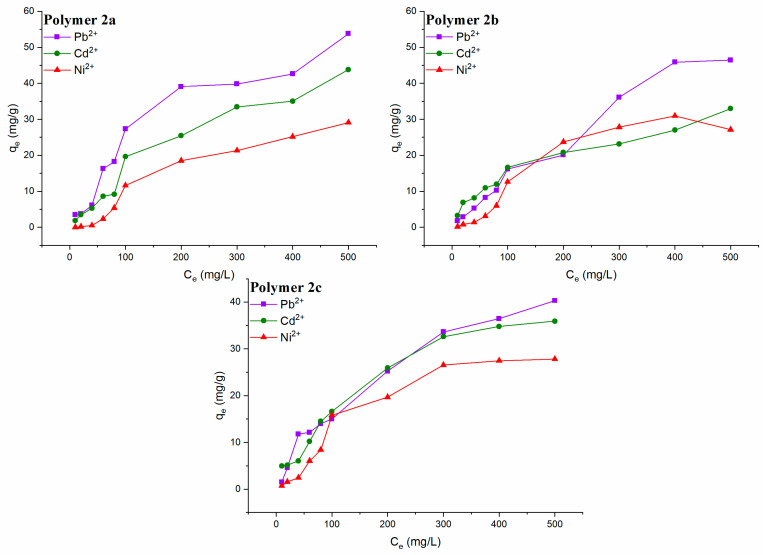
Amount of Pb^+2^, Cd^2+^ and Ni^2+^ adsorbed by the polymers **2a**, **2b** and **2c** as a function of the heavy metal solution concentration (*C_e_*).

The maximum equilibrium adsorption capacity (*q_e_*) of the three polymers as a function of the heavy metal solution concentration (*C_e_*) can be observed in Figure 8. The results show that the *q_e_* increases while *C_e_* also increases, where Pb^2+^ has the largest variation of the three divalent ions. The *q_e_* for polymer **2a** was found to be 53.78 mg/g for Pb^2+^, 43.80 mg/g for Cd^2+^ and 29.10 mg/g for Ni^2+^. The *q_e_* for polymer **2b** was found to be 46.45 mg/g for Pb^2+^, 32.95 mg/g for Cd^2+^ and 27.10 mg/g for Ni^2+^. The *q_e_* for polymer **2c** was found to be 40.31 mg/g for Pb^2+^, 35.93 mg/g for Cd^2+^ and 27.84 mg/g for Ni^2+^. The higher heavy metal adsorption capacity of polymer **2a** could be ascribed to the lower steric hindrance imparted by –SH groups to the polymer backbone compared to those of –SCH_3_ and –SCF_3_ groups in polymers **2b** and **2c**, respectively. The aforementioned, in conjunction with the higher hydrophilic character of the –SH groups could favor the water absorption by the polymer matrix, thus promoting the coordination between the polymer and the heavy metals during the ion adsorption process.

The correlation coefficient (*R*^2^) is useful to determine which model can best describe the equilibrium adsorption on the surface of polymers. The data with coefficient *R*^2^ < 0.92 were discarded due to poor fit of the data with the respective model, while other experimental data were adjusted acceptably (*R*^2^ > 0.95) to both models, so it is necessary to take more information, obtained from the theoretical fits, into account to suggest the adsorption behavior. The maximum theoretical adsorption capacity (*q_m_*) was used to decide which model best fit the experimental values. In this regard, the data acquired on the adsorption of Cd^2+^ in the polymer **2a**, Ni^2+^ and Cd^2+^ in the polymer **2b** and Pb^2+^ and Ni^2+^ in the polymer **2c**, suggest that the Langmuir model better describes the equilibrium adsorption, while the adsorption data for Pb^2+^ and Ni^2+^ in the polymer **2a**, Ni^2+^ in the polymer **2b** and Cd^2+^ in the polymer **2c**, suggest that the Freundlich model better describes the equilibrium adsorption. The parameters of the two models are shown in Table 3. The *K_F_* parameter shows the affinity of the ions for the polymer, while the constant *n* lets us know that the adsorption process is favorable due to the high adsorbent-adsorbate affinity, in addition to suggesting that the adsorption is carried out by the phenomenon of chemisorption [27,28,29]. We can see in Table 3 that *K_F_* for Ni^2+^ in polymers **2a** and **2b** is high, which suggests that the adsorption of the divalent ion should be higher than that of the other metals, but *n* < 1, which means that the adsorption of Ni^2+^ on the surface of these polymers is considerably weak, which may explain why the adsorption of Ni^2+^ is less than that of Pb^2+^ and Cd^2+^. The *K_L_* parameter also shows the affinity of the ions to the binding sites of the polymers.

### 3.3. Mass Effect

**Figure 9:**
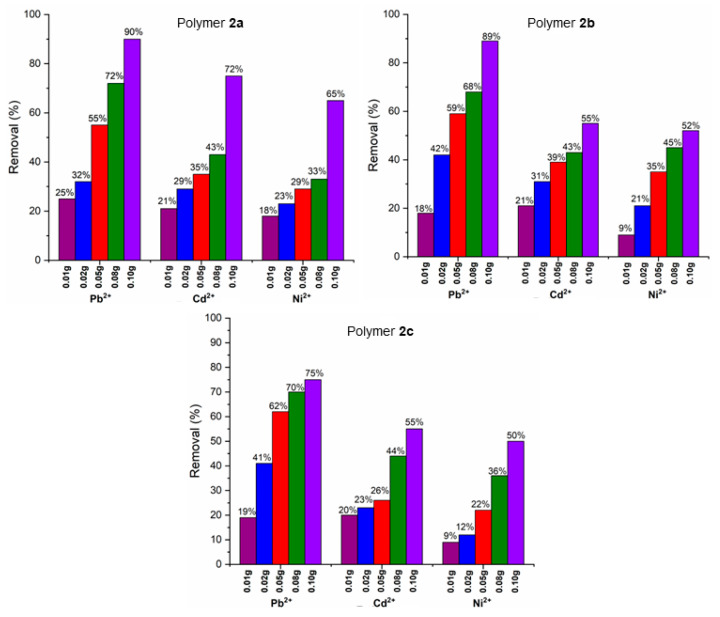
Percent removal of heavy metal ions versus the amount of polymer.

The equilibrium adsorption capacity behavior of the three polymers was studied by varying the amount of each polymer to adsorb heavy metals in an aqueous media. The heavy metal removal percentage against the mass of polymer is shown in Figure 9. A clear increase in adsorption capacity can be observed when more polymer is used, but this increase was not proportional to the increase in mass. The latter could be attributed to the ratio of mass to the surface area since the increase in surface area is not proportional to the increase in mass due to the physical properties of polymers. For instance, polymer **2a** had an increase of about 65% in the adsorption of Pb^2+^ when the amount of polymer was increased ten times; likewise, polymer **2b** had a 71% increase and polymer **2c** had a 56% increase in adsorption of the same ion. Polymer **2a** shows an increase of 54% and 47% for Cd^2+^ and Ni^2+^, respectively, while polymers **2b** and **2c** show a smaller increase in the adsorption of these divalent ions.

### 3.4. Multicomponent Adsorption

**Figure 10:**
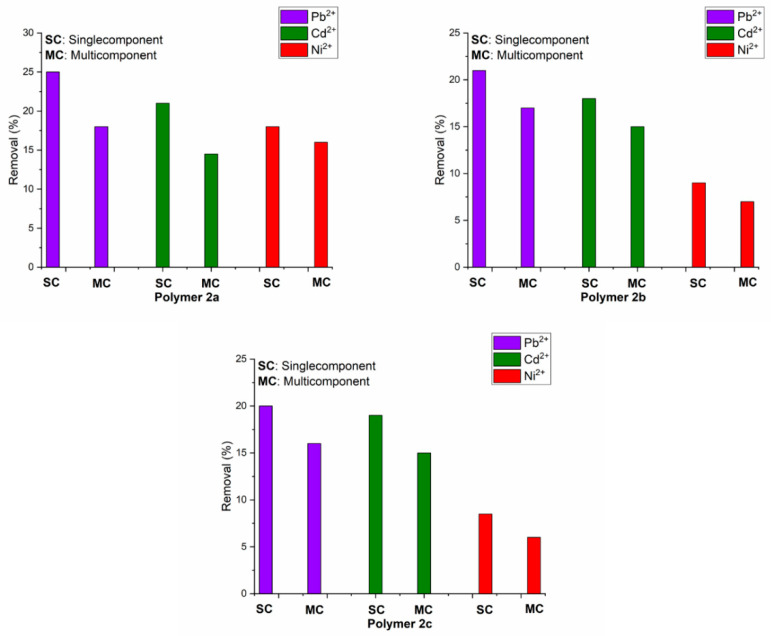
Percent removal of individual metals and in a ternary mixture of metals by the thiol-functionalized polymers.

The effect of ion competition was studied by adding each polymer to a mixture of Pb^2+^, Cd^2+^ and Ni^2+^. The results are shown in Figure 10, where it is possible to observe a decreasing trend in the removal of all ions. The decrease in the uptake of these heavy metal ions in the multicomponent mixture compared to that achieved in non-competitive circumstances could be due to the content for the same available adsorption sites on the thiol-functionalized polymer surface, the gradual active sites saturation and the shielding effect generated by the other competing metals. The removal efficiency order found is as follows: Pb^2+^ > Cd^2+^ > Ni^2+^; which is the same as that attained in the individual ions assessment. This trend in the removal efficiency presented by the polymers for Pb^2+^ over Cd^2+^ and Ni^2+^ can be ascribed to Pb^2+^ having a smaller hydration radius and hydration energy.

### 3.5. Polymer Surface

**Figure 11:**
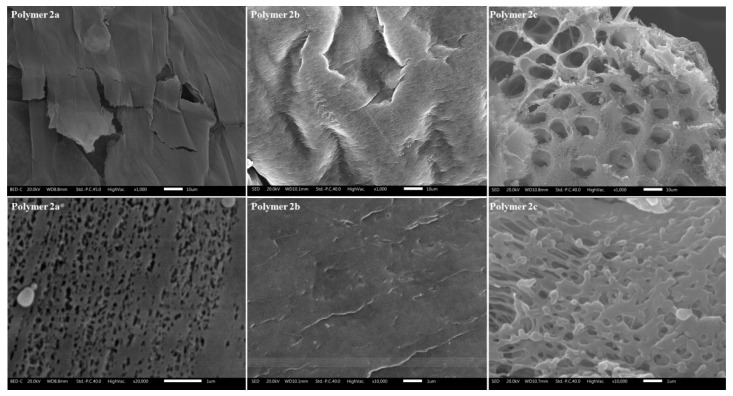
SEM images of the raw polymers bearing thiol moieties. Low image magnification (**top**) and high image magnification (**bottom**). The scales are 10 μm and 1 μm, respectively.

Topographic images of raw polymer samples were obtained using a scanning electron microscope and are presented in Figure 11. It is possible to see at low magnification (top) that polymers **2a** and **2b** have much smoother surfaces than that of polymer **2c**, where a porous structure prevails. In this sense, the bulky –CF_3_ groups are known to increase the polymer-free volume, thus promoting a material with microvoids. Using a higher resolution (bottom), it is possible to see that the surface of polymer **2a** is composed of a large number of microvoids that augment the surface area compared to polymer **2b**, which appears to maintain a regular morphology. The higher ion adsorption capacity of **2a** compared to **2b** could be attributed to its also higher surface area. Despite having an apparently increased surface area, it is likely that the hydrophobic character of the –CF_3_ groups in polymer **2c** restricts the water absorption by the polymer matrix, thus decreasing the coordination between the polymer and the heavy metals during the ion adsorption processes, which in turn is reflected in the lower adsorption capacity of all the polymeric materials examined here. More investigation is required to elucidate this subject.

Table 4 shows the maximum adsorption amounts of Pb^2+^, Cd^2+^ and Ni^2+^ for the thiol-functionalized polynorbornene dicarboximides reported in the present study and those of other polymeric adsorbents previously reported in the literature. It is seen that the outcomes found for the polymers bearing thiol pendant groups are comparable with those results of polymers functionalized with amide and sulfonic groups. It is worth mentioning that these results are even higher than those of some chelating polymers, which are adsorbents frequently employed for the heavy metal uptake from aqueous media. As the outcomes of this study indicate, the thiol group and its derivates show competitive adsorption capacities to be used effectively in the removal of Pb^2+^, Cd^2+^ and Ni^2+^ from aqueous solutions. This kind of polymer has several advantages over other materials; for instance, norbornene dicarboximide monomers are easily functionalized and obtained from economically accessible raw materials, thus leading to low-cost polymers. The polynorbornene dicarboximides bear hydrophobic cyclopentane rings that allow tailoring the mechanical performance, as well as hydrophilic imide moieties, which endow the polymer with ionic properties. This chemical structure favors the segregation of phases which in turn increases the adsorption area and the diffusion of the adsorbate within the material; thus, the balance in the structure and composition of the polymers could be used to tune the heavy metal removal capacity systematically. In addition, these polymers are easily processable and handled; therefore, they can be adapted to different environments to carry out adsorption in the most efficient way; that is, they can be used in the form of membranes, fibers or particles with a specific and homogeneous size. Likewise, these polymers are not soluble in water, so their separation, recovery and regeneration are very simple, thus increasing their economic viability.

## 4. Conclusions

Thiol-functionalized polynorbornene dicarboximides were successfully synthesized by ROMP and fully characterized by FT-IR, NMR, TGA and TMA, among others. The adsorptions kinetics of Pb^2+^, Cd^2+^ and Ni^2+^ in the polymers **2a**, **2b** and **2c** were investigated by batch adsorption experiments showing that the maximum uptake and the affinity in the thiol-functionalized polymers decreased in the order Pb^2+^ > Cd^2+^ > Ni^2+^ that could be ascribed to the higher atomic number and increasing heavy metals ionic radii. The adsorption kinetic data fit the pseudo-second-order model more efficiently, while the adsorption isotherms fit the Freundlich and Langmuir models. The adsorption capacity is attributed to the coordination that occurs between the divalent ions and the sulfur atom of the thiol groups. Moreover, the higher heavy metal uptake of polymer **2a** was attributed to the lower steric hindrance and higher hydrophilicity imparted by –SH groups to the polymer backbone compared to those of –SCH_3_ and –SCF_3_ groups in polymers **2b** and **2c**, respectively. According to the outcomes, polynorbornene dicarboximides containing thiol groups could potentially be used in ecosystem protection because of their effectiveness as heavy metal adsorbents, specifically to remove Pb^2+^, Cd^2+^ and Ni^2+^ from aqueous systems. The thiol-functionalized polymeric adsorbents reported in this work have higher uptakes than other adsorbents previously reported in the literature.

## Figures and Tables

**Table 1:** Physical parameters of the thiol-functionalized polymers.

Polymer	*T_g_* ^a^	*T_d_* ^b^	*ρ* ^c^	*d_spacing_* ^d^
	(°C)	(°C)	(g/cm^3^)	(Å)
**2a**	225	459	1.38	4.83
**2b**	216	409	1.23	4.88
**2c**	215	415	1.33	5.01

**^a^** glass transition temperature measured by DSC. **^b^** onset of decomposition temperature estimated by TGA. **^c^** density calculated by the flotation method in ethanol. **^d^** mean intersegmental distance determined by XRD.

**Table 2:** Kinetic parameters of the heavy metal uptake by the thiol-functionalized polymers.

Polymer	Metal	*q_t, exp_* (mg/g)	Pseudo-First Order			Pseudo-Second Order		
			*k_1_*	*q_e, the_*	*R* ^2^	*k_2_*	*q_e, the_*	*R* ^2^
			(min^−1^)	(mg/g)		(g/mg∙min)	(mg/g)	
**2a**	Pb^2+^	19.07	0.0027	9.04	0.69	0.0026	17.70	0.99
	Cd^2+^	15.49	0.0044	9.58	0.79	0.0008	16.66	0.98
	Ni^2+^	12.12	0.0016	5.22	0.68	0.0078	10.14	0.99
**2b**	Pb^2+^	15.98	0.0027	5.49	0.87	0.0646	15.20	0.99
	Cd^2+^	13.71	0.0020	2.38	0.67	0.2190	13.03	0.99
	Ni^2+^	10.95	0.0043	3.74	0.91	0.0756	10.81	0.99
**2c**	Pb^2+^	13.84	0.0057	2.97	0.83	0.1324	13.86	0.99
	Cd^2+^	11.12	0.0043	3.15	0.87	0.1017	11.03	0.99
	Ni^2+^	10.47	0.0023	3.16	0.47	0.1113	9.69	0.99

**Table 3:** Parameters of the Langmuir and Freundlich models for the adsorption of individual Pb^2+^, Cd^2+^ and Ni^2+^ ions by the thiol-functionalized polymers.

Polymer	Metal	*q_e, exp_* (mg/g)	Langmuir Isotherm			Freundlich Isotherm		
			*K_L_*	*q_m_*	*R* ^2^	*K_F_*	*n*	*R* ^2^
			(L/mg)	(mg/g)		(mg/g) (L/g)^1/*n*^		
**2a**	Pb^2+^	53.78	0.0082	39.06	0.84	1.91	1.29	0.92
	Cd^2+^	43.80	0.0043	44.84	0.98	1.24	1.21	0.97
	Ni^2+^	29.10	0.0034	3.85	0.90	267.73	0.65	0.92
**2b**	Pb^2+^	46.45	0.0038	45.87	0.98	4.43	1.14	0.97
	Cd^2+^	32.95	0.0134	27.70	0.97	1.12	1.83	0.97
	Ni^2+^	27.10	0.0080	1.91	0.90	89.38	0.73	0.94
**2c**	Pb^2+^	40.31	0.0015	108.69	0.95	2.24	1.31	0.93
	Cd^2+^	35.93	0.0240	21.32	0.74	1.03	1.67	0.95
	Ni^2+^	27.84	0.0017	41.66	0.98	1.27	1.00	0.94

**Table 4:** Maximum amounts of adsorption of Pb^2+^, Cd^2+^ and Ni^2+^ by the thiol-functionalized polymers studied in this work and other previously published adsorbent polymers.

Polymer	*q_e_* (Pb^2+^) (mg/g)	*q_e_* (Cd^2+^) (mg/g)	*q_e_* (Ni^2+^) (mg/g)
**2a**	53.7	43.8	29.1
**2b**	46.4	32.9	27.1
**2c**	40.3	35.9	27.8
5b ^*a*^ [19]	31.5	26.6	7.0
HDI-IC-PEHA Resin ^*b*^ [30]		69.4	26.4
Nafion 117 ^*c*^ [31]	58.0		64.0
XAD-4-NH-CH2-Cat ^*d*^ [32]	6.5	2.9	2.8

*^a^* Sulfonated ROMP-prepared polymer. *^b^* Polyamide bearing amine chelating moieties. *^c^* Sulfonic acid-containing fluoropolymer. *^d^* Chloromethylated polystyrene-divinylbenzene with dithiooxamide.

## Data Availability

The datasets used and/or analyzed during the current study are available from the corresponding author on reasonable request.
